# Clinical acupuncture therapy for children with allergic rhinitis: A protocol for systematic review and meta analysis

**DOI:** 10.1097/MD.0000000000024086

**Published:** 2021-01-22

**Authors:** Jun Li, Lanhua Liu, Lin Jiao, Kai Liao, LingnLing Xu, Xiaohong Zhou, Jun Xiong

**Affiliations:** The Affiliated Hospital of Jiangxi University of Traditional Chinese Medicine, Nanchang, China.

**Keywords:** acupuncture, allergic rhinitis, children, protocol, systematic review and meta-analysis

## Abstract

**Background:**

Allergic rhinitis (AR) in children has become a common clinical allergic disease, the incidence of which is increasing in pediatric. The side effects of the drug cause parents to worry about the health of their child. However, a large number of current clinical studies have shown that acupuncture therapy is effective in treating children with AR. Therefore, this systematic review aims to explore the safety and effectiveness of acupuncture in the treatment of AR in children.

**Methods:**

We will conduct a comprehensive literature search in Medline, PubMed, Cochrane Database of Systematic Reviews, Embase, Chinese Biomedical Literatures Database (CBM), China National Knowledge Infrastructure (CNKI), Wang Fang Database (WF), Chinese Scientific Journal Database (VIP) from inception to November 2020 without any language restriction. In addition, we will retrieve the unpublished studies and the references of initially included literature manually. Reviewers will identify studies, extract data, and assess the quality independently. The outcomes of interest include: total effective rate; the total nasal symptom score; Rhinitis quality of life questionnaire (RQLQ); Visual Analog Scale (VAS); Laboratory inspection indicators: the level of IgE, IL6, IL10 or TNF-α; Recurrence rate; adverse events. Randomized clinical trials will be collected, methodological quality will be evaluated using the Cochrane risk-of-bias assessment tool, and the level of evidence will be rated using the Grading of Recommendations, Assessment, Development and Evaluation approach. Meta-analysis will be performed using RevMan 5.4.0 software. The heterogeneity test will be conducted between the studies, *P* < .1 and *I*^2^ > 50% are the thresholds for the tests. We will utilize the fixed effects model or the random effects model according to the size of heterogeneity.

**Results:**

The results of this systematic review will provide a synthesis of current evidence of AR in children. We will report this result shortly.

**Conclusion:**

This study will explore whether or not acupuncture therapy can be used as one of the non drug therapies to prevent or treat allergic rhinitis in children.

**Trial registration number:**

INPLASY2020110053.

## 1 Introduction

Allergic rhinitis (AR) is an allergic disease dominated by type I allergic reaction on nasal mucosa and belongs to chronic non-infectious inflammation.^[1]^ In pediatrics, allergic rhinitis is one of the most common diseases which brings severe burden to their families and hinders the development of the society.^[2]^ It major symptoms are persistent nasal congestion, watery nose, paroxysmal sneezing, nasal itching, with or without decline or loss of smell;^[3]^ Apart from the nasal symptoms, it may also induce complications such as conjunctivitis, asthma, atopic dermatitis, sinusitis or serous otitis media^[4]^ that seriously affects the quality of life of children.

The occurrence of AR in children is related to many factors. The main external factors are includes: Air pollution, Climate change, domestic environment, parents smoking.^[5–8]^ At present, the underlying mechanism is still unclear. However, modern studies have found that childrens onset is mainly due to immunologic factors. Allergens are the most important substances that induce AR; Immunoglobulin E (IgE) antibodies produced by B lymphocytes are the most important sensitization pathways. The sensitization process of an individual is the inhalation of allergens in the nasal cavity, which binds to the IgE antibody on the mast cells on the nasal mucosa, then forms an antigen-antibody reaction in the nasal mucosa. The hypertrophy cell releases sensitizing substances such as histamine and leukotrienes (LTs), etc, after allergenic substances stimulate blood vessels and nerves on the nasal mucosa, finally cause corresponding symptoms.^[9–11]^ The incidence of patients with allergic rhinitis is increasing year by year, especially in developed countries. Moreover, 10% to 40% of adults and 2% to 25% of children in the world is affected by this disease. The International Study of Asthma and Allergies in Children (ISAAC) found a slight increase in the prevalence of children between 13 to 14 years (0.18% per year) and 6 to 7 years (0.17% per year);^[12,13]^ In the USA, the incidence of allergic rhinitis in children is 13%,^[14]^ The incidence rate of allergic rhinitis in urban areas of China is 9.8%, The morbidity of AR in children is more obvious, The number of children suffering from allergic rhinitis in cities is higher than in poor areas.^[15,16]^

Nowadays, the diagnosis and treatment of allergic rhinitis is becoming standardized. the clinical diagnosis must be clear about symptoms, signs, allergen screening and corresponding testing (anterior rhinoscopy or Nasal endoscopy, Allergen skin prick test and nasal mucosal provocation test, Local allergic rhinitis can be detected by nasal cavity challenge test and IgE test ).^[17]^ In this regard, treatment is divided into 2 clinical modes. On the one hand, non-drug therapy are nasal irrigation and lifestyle regulation; on the another hand, drug therapy are antihistamines, inhaled corticosteroids, sublingual immunotherapy (SLT) and antibiotic therapy^[18,19]^ which are used as major plans. However, the main purpose of the selective drug is to control the disease and its efficacy is also restricted by many factors. The drug itself also has limitations and side effects (Drowsiness, anxiety, irritability, palpitations, palpitation, etc).^[20,21]^ Based on this, some Medical scholars have proposed the use of traditional medicine as a supplementary therapy for allergic rhinitis or an alternative therapy that cannot tolerate medication.^[22]^

Acupuncture therapy is one of them. This therapy is now frequently used for the treatment of ENT diseases and has significant effects, It is gradually accepted and used by pediatrics.^[23,24]^ Some studies have found that acupuncture treatment of allergic rhinitis mainly regulates the balance of Th1/Th2 in the patient's serum, decreases the level of IgE, lessens the infiltration of inflammatory cells (eosinophils, mast cells) in the nasal mucosa and regulates the content of neuropeptides (substance P SP) to Relieve symptoms.^[25]^ Of course, some researchers have studied that acupuncture at different anatomical positions, depths and directions of the sphenopalatine ganglion can be effective in treating allergic rhinitis;^[26]^ It is also believed that acupuncture treatment of allergic rhinitis mainly improves the patients immunity and relieves the symptoms of discomfort.^[27]^ In addition, it originated from the Traditional Chinese Medicine (TCM) thinking that “A cure is as good as its own” and “keep healthy, do not be evil”. Acupuncture therapy has the characteristics of regulating qi-blood-yin-yang, preventing disease, having less side effects and being easily accepted by patients. Therefore, it is progressively popular all over the world.^[28,29]^

The use of systematic analysis is helpful to evaluate the effectiveness and credibility of clinical methods.^[30]^ So far, the number of patients with AR in children has gradually increased, the simplicity and low side effects of acupuncture therapy have attracted the attention of clinicians at home and abroad. Therefore, Randomized Controlled Trial (RCT) about acupuncture therapy in the treatment of allergic rhinitis in children has also increased. At present, there is still a lack of relevant systematic reviews in clinical practice. So this systematic review mainly describes the safety and effectiveness of acupuncture in the treatment of pediatric allergic rhinitis, in order to deal with adverse drug reactions or pediatric patients who wish to receive acupuncture therapy to make it better serve the clinic.

## 2 Methods and analysis

### 2.1 Study registration

This system evaluation plan has been registered on the International Prospective Register of Systematic Reviews (registration number INPLASY2020110053). You can check its authenticity on this website (https://inplasy.com/inplasy-2020-11-0053/). This article does not require ethical approval, and only analyzes the acupuncture treatment of children with AR that has been published in various databases. The protocol follows the Cochrane Handbook for Systematic Reviews and Meta-Analysis Protocol (PRISMA-P).^[31]^

### 2.2 Eligibility criteria

#### 2.2.1 Type of studies

We will comprehensively search the literature on acupuncture treatment in children with allergic rhinitis in Chinese and English databases. In addition, unpublished documents will be searched manually. The non-randomized controlled trials must be excluded.

#### 2.2.2 Types of participants

In this analysis of children with AR in children that the age must be less than 18 years old. Children who meet the internationally recognized diagnostic standard ARIA^[32]^ will be included, but the subjects gender, case, and ethnic origin are not limited Children with AR combined with other diseases (allergic asthma in children, allergic conjunctivitis in children) are going to excluded.

#### 2.2.3 Types of interventions

Children with AR in the test group must be treated with acupuncture as the main regimen (either in combination with other treatments or alone) and the control group must be treated with non-acupuncture therapy.

#### 2.2.4 Type of comparators

In the control group, the intervention means may include massage, medicine (Traditional Chinese medicine, western medicine), routine symptomatic treatment, immunotherapy etc, which acupuncture should not be used for the only requirement.

1. Acupuncture therapy vs no treatment;
2. Acupuncture therapy vs placebo;
3. Acupuncture therapy vs sham acupuncture;
4. Acupuncture therapy vs symptomatic or active treatment.

#### 2.2.5 Types of outcome measures

##### 2.2.5.1 Primary outcomes

Both the total effective rate and the total nasal symptom score (TNSS)^[33]^ were the main outcomes. The total nasal symptom score will be based on the symptoms (nasal congestion, watery nose, paroxysmal sneezing, nasal itching,) the patient presented.

##### 2.2.5.2 Secondary outcomes

1. Rhinitis quality of life questionnaire (RQLQ);^[34]^
2. Visual Analog Scale (VAS);
3. Laboratory inspection indicators: the level of IgE, IL6, IL10 or TNF-α;
4. Recurrence rate;
5. Adverse events.

### 2.3 Exclusion criteria

Before and after experiment research; Acupuncture is present in the control group; Repeated literature, theoretical discussion and review literature, nursing literature, animal experimental research, etc.

### 2.4 Search strategy

We will search the following databases: PubMed, Cochrane Database of Systematic Reviews, Embase, Chinese Biomedical Literatures Database (CBM), China National Knowledge Infrastructure (CNKI), Wang Fang Database (WF), and Chinese Scientific Journal Database (VIP) from their inception to November 2020. The main subject terms searched: “acupuncture”, “children”, “allergic rhinitis” Pubmed's search strategy is shown in Table 1; other database search strategies will be adjusted according to each database.

**Table 1:** Search Strategy (PubMed).

Order	Strategy
#1	Search “Allergic rhinitis” [Mesh] OR“ Allergic rhinitis seasonal” [Mesh] OR“ Allergic rhinitis perennial”[Mesh]
#2	Search “Allergic rhinitis” [Ti/Ab] or “rhinallergosis” [Ti/Ab] or “hypersensitive rhinitis” [Ti/Ab] or “anaphylactic rhinitis” [Ti/Ab] or “perennial allergic rhinitis” [Ti/Ab] or “pollinosis” [Ti/Ab] or “seasonal allergic rhinitis” [Ti/Ab] or “nasal allergy” [Ti/Ab] “allergic rhinoconjunctivitis” or ”hay fever” [Ti/Ab] or ”AR” [Ti/Ab]
#3	#1 OR #2
#4	Search “Acupuncture” [Mesh] OR “Acupuncture Therapy” [Mesh] OR “Acupuncture, Ear” [Mesh] OR “Acupuncture Points” [Mesh]
#5	Search “acup∗” [Ti/Ab] or “need∗” [Ti/Ab] or “Acupuncture Therapy” [Ti/Ab] or “Acupuncture treatment ” [Ti/Ab] or “Pharmacopuncture” [Ti/Ab] or “Meridians” [Ti/Ab] or “ Acupuncture Points” [Ti/Ab] or “electropuncture ” [Ti/Ab]“ Ear acupuncture” or ”Fire needle ” [Ti/Ab] or ”Warming needle” [Ti/Ab]
#6	#4 AND #5
#7	Search “Restriction of ages:birth-18 years”
#8	Search “child∗” [Ti/Ab] or “” [Ti/Ab] or “pediatric” [Ti/Ab] or “infant ” [Ti/Ab]
#9	#7 OR #8
#10	Search “Randomized controlled trial” [MeSH] or “controlled clinical trial” [MeSH]
#11	Search “Randomized controlled trial” [Ti/Ab] or “clinical trial” [Ti/Ab] or “randomized” [Ti/Ab]
#12	#10 OR #11
#10	#3 AND #6 AND #9 AND #12

### 2.5 Process of selection

**Figure 1:**
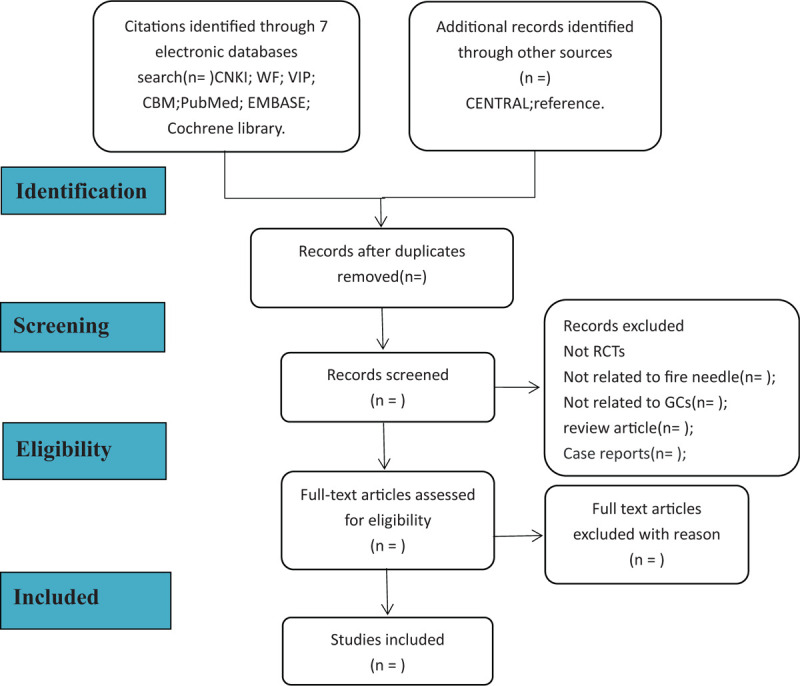
Flowchart of literature selection.

We will deal with the included literature in the following way and the specific operation is as follows: Firstly, according to the selected topic, the retrieved literature is imported into note Express 3.0 in the document manager in the correct retrieval way, and remove the duplicate published article in the document manager; Then, by reading the title and abstract of the essay one by one, weed out the article irrelevant to this research; Then, download the remaining articles in sequence and read the full text; Finally, according to the inclusion and exclusion criteria required in this article, the final paper is determined;

For this operation, 2 researchers (Kai Liao, Lingn Ling, Xu) strictly follow this procedure. If they disagree, consult the third evaluator (Jun Li) for negotiation. The included article process is shown in Figure 1.

#### 2.5.1 Information extraction

In the final article, 2 researchers (Kai Liao, Lingn Ling, Xu) independently extracted information and entered it into word 2010. After the end, they were cross-checked. If there are any doubts, they can contact the third evaluator (Jun Li) Negotiate processing to ensure the accuracy of the information. The extracted information includes: title, Author, year of publication, sample size, intervention measures and treatment course, etc. When some important information is missing in the input data of some articles, contact the author by phone or email.

#### 2.5.2 Methodological quality evaluation

Use the Cochrane Reviewer's Handbook 5.0 to evaluate the quality of the final selected literature and assess the risk of bias.^[35]^ The main contents are: Random method; allocation concealment; implementation of blind method; blindness of the outcome rater; completeness of the result data; selective reporting of results; other biases. Each of the above 3 items contains “yes”, “no” and “unclear”. The 2 assessors (Kai Liao, Lingn Ling, Xu) need to evaluate the options that meet the conditions. If there is any dispute among them, they can discuss and deal with a third party (Jun Li).

### 2.6 Data synthesis

#### 2.6.1 Quantitative data analysis

Enumeration data will be represented by odds ratio (OR) and 95% confidence interval (CI), measurement data are represented by weighted mean difference (WMD) and 95% confidence interval (CI) or standardized mean difference (SMD) should be used when the units were not unified.

#### 2.6.2 Heterogeneity analysis

When performing heterogeneity test, use *I*^2^ test. When *P* > .1 and *I*^2^ < 50%, use fixed effects model; otherwise, use random effects model. Sensitivity analysis will be used if the heterogeneity is large. If there is substantial heterogeneity, it can be analyzed descriptively. Use Review Manager 5.4.0 line inverted funnel chart to qualitatively analyze publication bias.

#### 2.6.3 The publication bias

If the number of remaining articles is more than 10, we can use Review Manager 5.4.0 line inverted funnel chart to qualitatively analyze the publication bias. The graph shows the approximate shape which represents the publication bias.

#### 2.6.4 Subgroup analyses

If there is large heterogeneity, we will conduct subgroup analysis based on different control measures.

#### 2.6.5 Sensitivity analysis

The sensitivity analysis should be performed to assess the reliability of the meta-analysis; analysis software uses STATA 14.0 software for sensitivity analysis.

## 3 Discussion

This review is the first review of the current modern literature on acupuncture in the treatment therapy of AR in children, the purpose of which is to find the beneficial evidence for acupuncture therapy for allergic rhinitis in children. Based on the discussion part of the article, it will be described in the following aspects:

1. Pathogenesis of AR in children with traditional Chinese medicine (TCM);
2. Advantages, disadvantages and mechanism of acupuncture therapy in the treatment of AR in children;
3. Horizontal comparison with other treatment methods or viewpoints;
4. Interpret the results;
5. conclusion;

Acupuncture therapy has a history of thousands of years; it has been used as a supplement and substitute therapy by doctors at home and abroad. The treatment of pediatric diseases is gradually being promoted. Acupuncture is a boon for pediatric patients.^[36]^ Acupuncture therapy has become the dominant disease of allergic rhinitis that intervention may play its role by regulating various pathways and activating various mediators.^[37,38]^ At present, there is no systematic scientific evaluation of acupuncture in children with AR, so this article aims to provide evidence-based medical opinions on the safety of acupuncture in the treatment of children with AR. Of course, this study still has certain limitations: first, the quality of the article is not high, which may affect the evaluation results; second, the essay has Chinese and English language restrictions, which may lead to incomplete literature retrieval; third, there may be the author of the article can not be contacted, resulting in incomplete data results. Therefore, for more high-quality randomized controlled trials and research mechanisms are needed to confirm its effectiveness, so as to more objectively evaluate the safety and effectiveness of acupuncture in the treatment of AR in children.

## Author contributions

**Conceptualization:** Jun Li.

**Data curation:** Kai Liao, Lingling Xu.

**Formal analysis**: Jun Li, Lanhua Liu.

**Investigation:** Lin Jiao.

**Methodology:** Xiaohong Zhou.

**Project administration**: Jun Xiong.

**Software:** Kai Liao.

**Supervision**: Jun Xiong.

**Validation**: Jun Xiong.

**Writing – original draft:** Jun Li, Kai Liao, LingnLing, Xu.

**Writing – review & editing:** Lanhua Liu, Jun Xiong.
